# Supplementary figures and images for: Short communication: miRNA122 interrogation via PCR-Free method to track liver recovery
Source: PLoS One. 2025 May 30;20(5):e0324858. doi: 10.1371/journal.pone.0324858 (PMC12124506; doi:10.1371/journal.pone.0324858)

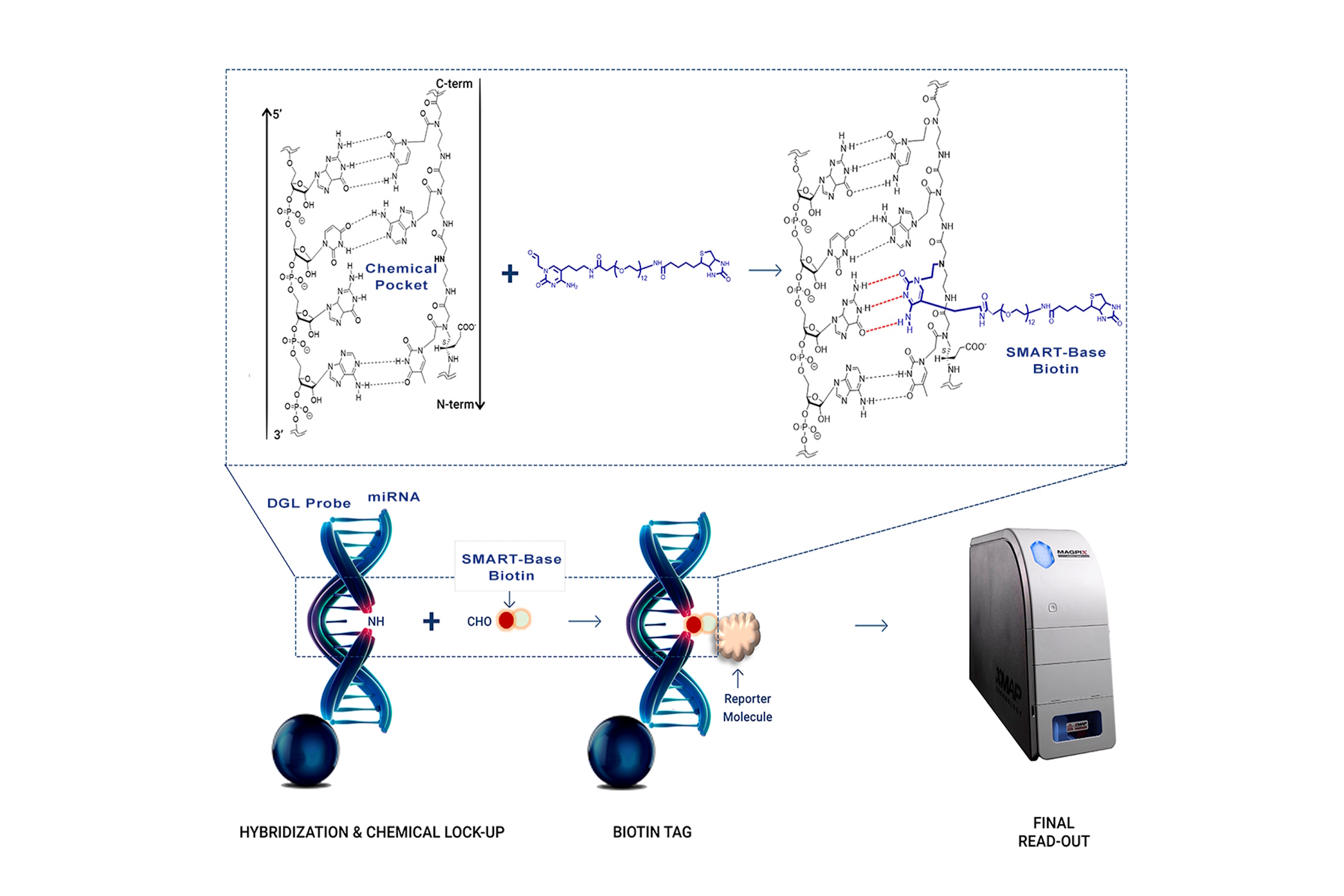

Supplement: S1 Fig — The DGL Probe captures the complementary single stranded nucleic acid (miRNA) sequence, forming the Chemical Pocket. Once the target miRNA is fully hybridized, the SMART-Base Biotin is incorporated and covalently linked to the backbone of the DGL Probe, resulting in the Chemical Lock up. The duplex is then detected using a reporter molecule, such as Streptavidin Phycoerythrin, which specifically recognizes the biotin tag. The final read-out is performed using the Luminex platform. (Anal. Methods, 2023,15, 6139–6149). (JPG) [file pone.0324858.s001.jpg]

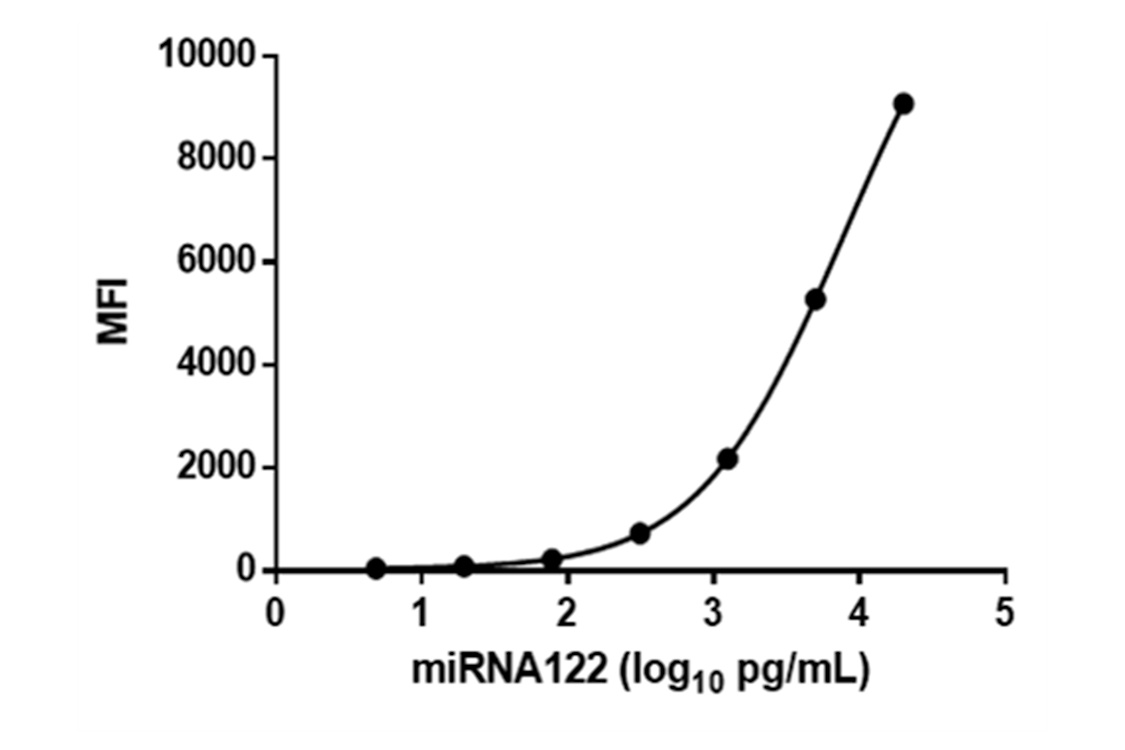

Supplement: S2 Fig — Calibration curve was constructed by utilizing a 5PL non-linear regression model, where MFI Average values were plotted against the logarithm of phase 10 of concentration of synthetic oligonucleotide mimicking miRNA122. Seven-point concentrations were tested by spiking in commercially available serum matrix (MP Biomedicals™, Cat. Number 11465055). Concentrations were respectively 20000.00, 5000.00, 1250.00, 312.50., 78.13, 19.53 and 4.88 pg/mL. Non-spiked-in serum was used as negative control. Each measurement was conducted in duplicate. (JPG) [file pone.0324858.s002.jpg]
