# Supplementary material for: Short communication: miRNA122 interrogation via PCR-Free method to track liver recovery
Source: PLoS One. 2025 May 30;20(5):e0324858. doi: 10.1371/journal.pone.0324858 (PMC12124506; doi:10.1371/journal.pone.0324858)
Supplement: S1 Table — (PDF) [file pone.0324858.s003.pdf]

**Parameter**	**Result**
Sensitivity; LOD	10.22 pg/mL
Sensitivity; LLOQ	19.53 pg/mL
Precision	3.36 %
Accuracy	99.80 %

This table was generated using the calibration curve shown in S2 Fig, along with the corresponding data provided on page 4 of S1 Text.

Sensitivity (LOD) was calculated as 3 times the standard deviation (SD) plus the average of the blank measurements.

Sensitivity (**LLOQ**) was calculated as 10 times the SD plus the average of the blank measurements.

Precision was assessed by calculating the standard deviation or coefficient of variation (CV) across multiple replicates at each concentration point, and then taking the average CV across all concentrations.

Accuracy was determined as the average recovery percentage across all tested concentrations.
