# Supplementary material for: Short communication: miRNA122 interrogation via PCR-Free method to track liver recovery
Source: PLoS One. 2025 May 30;20(5):e0324858. doi: 10.1371/journal.pone.0324858 (PMC12124506; doi:10.1371/journal.pone.0324858)
Supplement: S2 Table — (PDF) [file pone.0324858.s004.pdf]

**Parameter**	**miRNA122 vs ALT**	**miRNA122 vs AST**	**miRNA122 vs TBL**
**Pearson r**			
r	0.8150	0.7895	0.2646
95% confidence interval	0.5497 to 0.9310	0.4977 to 0.9208	-0.2866 to 0.6842
R square	0.6643	0.6234	0.07001
**P value**			
P (two-tailed)	< 0.0001	0.0002	0.3406
P value summary	****	***	ns
Significant? (alpha = 0.05)	Yes	Yes	No
