# Supplementary material for: Short communication: miRNA122 interrogation via PCR-Free method to track liver recovery
Source: PLoS One. 2025 May 30;20(5):e0324858. doi: 10.1371/journal.pone.0324858 (PMC12124506; doi:10.1371/journal.pone.0324858)
Supplement: S1 Text — (PDF) [file pone.0324858.s005.pdf]

Raw Data Used to Generate Fig 1(a)

(Values in the grey column were used to construct the Fig 1a)

Group	Visit	Avg. Result (pg/mL)	Result (pg/mL)	Result SD	Result CV	Avg. MFI	MFI	MFI CV	MFI SD	n
**Patient 1**	**1**	49,15	44,91<br>53,39	6,00	12,2%	160,00	150,00<br>170,00	8,8%	14,14	2
	**2**	24,94	21,13<br>28,76	5,40	21,6%	101,50	92,00<br>111,00	13,2%	13,44	2
	**3**	-	-<br>-	-	-	35,00	35,00<br>35,00	0,0%	0,00	2
**Patient 2**	**1**	57,47	54,68<br>60,27	3,95	6,9%	179,50	173,00<br>186,00	5,1%	9,19	2
	**2**	33,27	29,57<br>36,97	5,23	15,7%	122,00	113,00<br>131,00	10,4%	12,73	2
	**3**	4,92	4,74<br>5,10	0,25	5,2%	49,50	49,00<br>50,00	1,4%	0,71	2
**Patient 3**	**1**	81,51	80,85<br>82,18	0,94	1,2%	234,50	233,00<br>236,00	0,9%	2,12	2
	**2**	49,99	51,69<br>48,29	2,40	4,8%	162,00	166,00<br>158,00	3,5%	5,66	2
	**3**	6,00	6,18<br>5,82	0,26	4,3%	52,50	53,00<br>52,00	1,3%	0,71	2
**Patient 4**	**1**	38,64	36,56<br>40,71	2,94	7,6%	135,00	130,00<br>140,00	5,2%	7,07	2
	**2**	33,88	30,80<br>36,97	4,36	12,9%	123,50	116,00<br>131,00	8,6%	10,61	2
	**3**	-	-<br>-	-	-	40,00	40,00<br>40,00	0,0%	0,00	2
**Patient 5**	**1**	58,33	56,82<br>59,83	2,13	3,7%	181,50	178,00<br>185,00	2,7%	4,95	2
	**2**	55,11	57,68<br>52,54	3,64	6,6%	174,00	180,00<br>168,00	4,9%	8,49	2
	**3**	30,80	32,02<br>29,57	1,73	5,6%	116,00	119,00<br>113,00	3,7%	4,24	2
**Patient 6**	**1**	18,75	18,75<br>18,75	0,00	0,0%	86,00	86,00<br>86,00	0,0%	0,00	2
	**2**	9,33	8,39<br>10,26	1,32	14,2%	61,50	59,00<br>64,00	5,7%	3,54	2
	**3**	-	-<br>-	-	-	33,00	33,00<br>33,00	0,0%	0,00	2
**Patient 7**	**1**	7,84	8,39<br>7,28	0,78	10,0%	57,50	59,00<br>56,00	3,7%	2,12	2
	**2**	-	-<br>-	-	-	36,00	36,00<br>36,00	0,0%	0,00	2
	**3**	-	-<br>-	-	-	37,00	39,00<br>35,00	7,6%	2,83	2
**Patient 8**	**1**	8,74	8,74<br>8,74	0,00	0,0%	59,50	61,50<br>61,50	0,0%	0,00	2
	**2**	-	-<br>2,63	-	-	42,50	42,00<br>43,00	1,7%	0,71	2
	**3**	-	-<br>-	-	-	37,50	38,00<br>37,00	1,9%	0,71	2

Raw Data Used to Generate Fig 1(b–d)

Biochemical parameter	Patient nº							
	**1**	**2**	**3**	**4**	**5**	**6**	**7**	**8**
**Visit 1**								
TBL mg/dL	0,69	11,43	2,94	22,71	28,4	5,72	1,19	13,75
AST xULN	9,55	15,15	8,53	21,8	15,93	21,5	17,55	25,75
ALT xULN	8,25	15,9	6,68	25,3	14,4	36,83	24,2	33,22
**Visit 2**								
TBL mg/dL	0,54	1,79	2,43	30,58	28	5,56	1,13	7,2
AST xULN	0,75	0,98	12,75	19,83	16,08	17,1	9,9	13,2
ALT xULN	4,73	1,68	9,38	12,98	10,35	24,75	15,55	21,15
**Visit 3**								
TBL mg/dL	0,39	0,4	2,2	5,41	1,39	1,52	0,7	0,6
AST xULN	0,58	No data	0,95	10,43	No data	2,5	1,87	No data
ALT xULN	0,73	0,53	0,87	10,38	0,9	4,95	2,87	0,72

Raw Data Used to Generate Fig 2 and S2 Table

	Visit	miRNA122	ALT	AST	TBL
Patient 1	1	49,15	33,22	25	13
	2	24,94	21,15	13	7
	3		0,72		
Patient 2	1	57,47	24,2	17	1
	2	33,27	15,55	9	1
	3	4,92	2,87	1	
Patient 3	1	81,51	36,83	21	5
	2	49,99	24,75	17	5
	3	6	4,95	2	1
Patient 4	1	38,64	14,4	15	28
	2	33,88	10,35	16	28
	3		0,9		1
Patient 5	1	58,33	25,3	21	22
	2	55,11	12,98	19	30
	3	30,8	10,38	10	5
Patient 6	1	18,75	6,68	8	2
	2	9,33	9,38	12	2
	3		0,87		2
Patient 7	1	7,84	15,9	15	11
	2		1,68		1
	3		0,53		
Patient 8	1	8,74	8,25	9	
	2		4,73		
	3		0,73		

Raw Data Used to Generate S2 Fig

(The precision and accuracy highlighted in grey are also reported in S1 Table.)

Expected (pg/mL)	Avg. Result (pg/mL)	Result (pg/mL)	Result SD	Result CV	Avg. MFI	MFI	MFI CV	MFI SD	Avg. Recovery	Recovery	n
20000,00	19743,99	19700,48	61,53	0,3%	9077,00	9181,00	1,6%	147,08	98,7%	98,5%	2
		19787,50				8973,00				98,9%	
5000,00	4858,67	5085,55	320,85	6,6%	5275,50	5405,00	3,5%	183,14	97,2%	101,7%	2
		4631,79				5146,00				92,6%	
1250,00	1250,80	1272,05	30,05	2,4%	2179,00	2207,00	1,8%	39,60	100,1%	101,8%	2
		1229,55				2151,00				98,4%	
312,50	323,92	336,49	17,77	5,5%	729,75	753,50	4,6%	33,59	103,7%	107,7%	2
		311,36				706,00				99,6%	
78,13	75,76	76,86	1,56	2,1%	221,50	224,00	1,6%	3,54	97,0%	98,4%	2
		74,66				219,00				95,6%	
19,53	19,74	19,94	0,28	1,4%	88,50	89,00	0,8%	0,71	101,1%	102,1%	2
		19,54				88,00				100,1%	
4,88	4,92	5,10	0,25	5,2%	49,50	50,00	1,4%	0,71	100,8%	104,4%	2
		4,74				49,00				97,1%	
0,00	-	-	-	-	35,00	37,00	8,1%	2,83	-	-	2
		-				33,00				-	
			Precision	3,35%					Accuracy	99,80%	
