# Supplementary material for: Short communication: miRNA122 interrogation via PCR-Free method to track liver recovery
Source: PLoS One. 2025 May 30;20(5):e0324858. doi: 10.1371/journal.pone.0324858 (PMC12124506; doi:10.1371/journal.pone.0324858)
Supplement: Attachment — Submitted filename: Response to Reviewers.docx [file pone.0324858.s007.docx]

**Response to reviewers**

Below we provide detailed responses to each of the points raised by the two reviewers. The reviewers' comments are in black, followed by our responses and corresponding actions in Azzure.

**Reviewer #1**

The manuscript titled "Short communication: miRNA122 interrogation via PCR-free method to track liver recovery" presents a novel approach to diagnosing and monitoring acute liver injury (ALI) using miRNA122 as a biomarker, measured with the Dynamic Chemical Labeling (DCL) PCR-Free method on Luminex platforms. The study's strengths include its innovative method, direct applicability in clinical settings, and validation of miRNA122 as a potential liver-specific biomarker. However, there are several points that require clarification and expansion to improve the rigor and completeness of the study.

Questions to the authors:

**Question # 1:** Given the small sample size of eight patients, could you please provide a statistical power analysis or rationale for this cohort size? How might the limited sample size impact the generalizability of your findings to broader patient populations with acute liver injury (ALI)? The absence of a control group (e.g., healthy individuals or patients with other liver conditions) makes it difficult to assess the specificity of miRNA122 as a biomarker for ALI.

Reply # 1: We appreciate the reviewer’s insightful comments regarding the sample size and the absence of a control group. We acknowledge these limitations and would like to clarify the rationale behind our study design. This investigation was conceived as a **proof-of-concept study to evaluate the feasibility of using the DCL PCR-Free method for miRNA-122 quantification in ALI patients**. Given the exploratory nature of the study, the cohort size was not determined based on formal power calculations but rather on the need to establish technical feasibility before proceeding to larger validation studies.

Despite the small sample size, our findings demonstrate strong correlations between miRNA-122 and conventional liver biomarkers (ALT and AST), reinforcing its potential as a biomarker for ALI monitoring. While a larger cohort would allow for more robust statistical conclusions, the observed consistency across our data provides preliminary support for the biological relevance of miRNA-122 in ALI.

Regarding the concern about specificity, we agree that incorporating a control group, such as healthy individuals or patients with other liver conditions, would enhance the assessment of miRNA-122's specificity for ALI. While this was beyond the scope of our initial study, we anticipate that future research will address this important aspect.

To further support the potential of the assay, we reference an **independent study** in which the DCL PCR-Free method has been successfully employed for the identification of the LiverAce Biomarker Panel, comprising miRNA-122 alongside Keratin 18 (both total and caspase-cleaved forms). These biomarkers are known to play key roles in diagnosing and understanding acute liver injury, providing insights into hepatocellular injury, inflammation, cytotoxicity, disease severity, and prognosis.

In this independent study, the assay was applied to a cohort of **15 ALI patients** and **10 healthy volunteers as a control group**, enabling a direct comparison between diseased and non-diseased individuals. The results demonstrated a clear differentiation between ALI and healthy controls based on the biomarker panel, with significantly elevated levels of miRNA-122, total K18, and caspase-cleaved K18 (ccK18) in liver injury patients compared to the control group. These findings provide further validation for the specificity and clinical utility of miRNA-122 as a biomarker for liver injury.

In summary, while our study was limited in sample size and lacked a control group, our findings, together with independent validation data, strongly support the feasibility of miRNA-122 quantification using the DCL PCR-Free method. **Future studies** will focus on **expanding the cohort size and incorporating appropriate controls** to further validate miRNA-122’s diagnostic specificity in ALI.

Evaluation of DCL PCR-Free method on Luminex in ALI patients and healthy controls. A) microRNA122 (miRNA122), B) Cytokeratin 18 (K18), and C) Caspase-cleaved Cytokeratin 18 (ccK18)—are presented. The y-axis indicates the Mean Fluorescence Intensity (MFI) as measured by the Luminex MAGPIX platform. Samples were provided by the biospecimen supplier Boca Biolistics’ biobank.

**Question # 2:** The Dynamic Chemical Labeling (DCL) PCR-Free method shows promise, particularly in its high sensitivity and precision. However, could you elaborate on any potential limitations or biases inherent in DCL technology, such as specificity issues with similar miRNAs or interference from other serum components?

Reply # 2: We appreciate the reviewer’s question regarding potential limitations of the DCL PCR-Free method. DCL achieves high specificity for miRNA122 through a three-step proofreading process.

1. First, Watson-Crick base pairing ensures that only miRNA122 binds to the DGL probe.
2. Second, the probe contains an abasic site that prevents mislabeling; even a single mismatch destabilizes the duplex and blocks the reaction.
3. Third, only perfectly matched miRNA122 undergoes biotin incorporation, enabling single-nucleotide discrimination.

This intrinsic proofreading mechanism eliminates cross-reactivity with similar miRNAs, ensuring unmatched sequence specificity.

Regarding interference from serum components, DCL is resistant to enzymatic inhibitors such as heparin, which commonly affect PCR-based assays. The StabilTech buffer effectively releases miRNA122 from extracellular vesicles and protein complexes, improving recovery while preventing degradation. The use of PNA-based DGL probes further enhances stability, as these are completely resistant to nucleases and proteases. Additionally, the magnetic bead-based capture system isolates miRNA122, removing non-specific interactions and ensuring clean detection.

Unlike traditional PCR-based methods, DCL is not affected by enzymatic biases or inhibitors, making it highly suitable for direct miRNA quantification in serum. Its high specificity and robustness ensure reliable detection of miRNA122 with minimal interference from similar sequences or other serum components.

**Question # 3:** In your results section, miRNA122 levels showed a significant correlation with ALT and AST, but not with TBL. Could you discuss the potential biological implications of these findings? How might the kinetic profiles of these biomarkers differ in the context of liver injury progression and recovery? Why were only three time points chosen? Would higher temporal resolution provide more insights into the dynamics of miRNA122 during liver injury and recovery?

**Reply # 3**: We appreciate the reviewer’s insightful observations.

**The different correlations between miRNA122 and traditional biomarkers** is likely due to differences in kinetic profiles of these biomarkers during liver injury and recovery. ALT and AST are hepatocellular enzymes released into the bloodstream upon liver injury, whereas TBL is a functional biomarker reflecting degree of liver injury. In addition, TBL levels are influenced by multiple factors, including level of inflammation and bile excretion capacity, which may explain the lack of correlation.

**Regarding the differences in kinetic profiles:** ALT, AST, and miRNA122 typically exhibit an early rise following liver injury, followed by a progressive decline during recovery. Conversely, TBL levels generally peak later and reflect impaired liver function, following a more variable kinetic pattern depending on the severity of cholestasis and hepatic dysfunction. The observed lack of correlation between miRNA122 and TBL supports the idea that miRNA122 is more closely associated with hepatocyte integrity than with liver excretory function.

**Regarding the rationale for selecting three time points**: The three chosen time points were strategically selected to capture different phases of liver injury: detection of the acute episode, which is close related to the acute phase (day 1); an intermediate phase (day 7), and the recovery phase (≥30 days). The 30-day time point was particularly important, as clinical experience suggests that by this stage, patients with acute liver injury often exhibit significant clinical improvement. This time frame allowed us to evaluate whether miRNA122 levels continued to decrease alongside traditional biomarkers, reinforcing its potential as a monitoring tool. While a longer follow-up period would have provided stronger evidence for a parallel decrease in AST, ALT and miR-122 values, many of the patients had normalized liver profile values at visit 3 and therefore were not scheduled for further follow-up visits by their physicians.

**Question # 4:** Your study predominantly groups all ALI cases together without stratification by the underlying cause of liver injury. Are there plans to analyze how different etiologies (e.g., drug-induced, viral hepatitis, alcohol-related) might influence miRNA122 levels? Did the authors consider including healthy controls or patients with other liver conditions to compare miRNA122 levels and validate the method's liver specificity?

Reply # 4. We appreciate the reviewer’s suggestion regarding stratification by etiology and the inclusion of controls.

**Stratification by etiology**: While our study aimed to evaluate miRNA122 dynamics in ALI as a whole, we acknowledge that different causes (e.g., drug-induced, viral, or alcohol-related) may influence biomarker profiles. Future analyses with larger cohorts will explore how etiology impacts miRNA122 kinetics, which could provide additional insights into its diagnostic and prognostic value.

**Inclusion of controls**: This study focused on ALI patients to assess miRNA122 as a monitoring tool. However, we recognize the importance of validating its liver specificity by comparing it with healthy individuals and patients with other liver conditions. Expanding the study to include these groups would further support the robustness of miRNA122 as a liver biomarker, and we plan to address this in future research.

**Question # 5:** Given the involvement of several authors who hold positions in DESTINA Genomica SL, which markets the assay used in this study, how do you address potential conflicts of interest? Could these relationships have influenced the study design, data interpretation, or reporting?

Reply # 5: We have disclosed in our submission not only the involvement of the company's employees, but also the involvement of the company's co-inventor and co-founder, Prof Diaz-Mochon, who has a long academic career. The DESTINA R&D team involved in this manuscript consists of a group of PhDs with a strong publication background in various fields. This team has co-authored more than 20 peer-reviewed publications on DCL technology with top academic and clinical collaborators. None of these publications have been challenged. Whilst we understand the concern of the reviewer, we believe that our credibility and approach is well deserved and recognised. The company's motto is Science First. Furthermore, the clinical co-authors of the manuscript, led by Prof. Raul Andrade and M. Isabel Lucena, together with Salvatore Pernagallo, were primarily responsible for the study design, data interpretation and reporting. DESTINA provided the assay reagents, protocols and performed the assays. Prof. Andrade's group is well known in the field of liver injury.

**Question # 6:** The study tracks patients over three visits within a 30-day period post-ALI recognition. Could you discuss the potential for extending this follow-up period to evaluate the long-term predictive value of miRNA122 for chronic liver outcomes or recurrence of liver injury?

Reply # 6: We appreciate the reviewer’s interesting suggestion. Our study focused on short-term monitoring of ALI recovery, as the 30-day time point aligns with significant clinical improvement in most patients. We acknowledge that a longer follow-up period could provide valuable insights into whether miRNA122 levels serve as predictors of chronic liver outcomes or the risk of recurrent liver injury. However, the proportion of patients with acute liver injury, who develop chronic liver injury despite a significant decrease in liver profile elevations 30 days from detection, is small. In fact, all of the patients included in the current study presented a normal liver profile 30 days after detection or close thereafter.

**Question # 7:** Are there specific technological improvements that could be made to enhance the DCL PCR-Free method? For instance, could modifications in probe design or detection sensitivity further refine the specificity and robustness of this method? How does the DCL PCR-Free method ensure specificity for miRNA122 in the presence of other circulating miRNAs or nucleic acids?

Reply # 7: The DCL PCR-Free method is platform agnostic and can therefore be used with other detection platforms. One way to improve the technology is to use other ultrasensitive platforms that could increase the sensitivity of the assay. Ultimately, the DCL technology is as sensitive as the platform it is run on. As mentioned in our answer to your question #2, the high specificity of the technology is based on this two-step process that avoids false positive results.

**Question # 8:** Could you provide more detail on the statistical methods used to assess correlations and their appropriateness for this type of longitudinal biomarker data? Were there any differences in miRNA122 levels based on demographic factors (e.g., age, sex) or the severity/type of liver injury? Did you adjust for potential confounders such as medication history, comorbidities, or other liver biomarkers in your correlation analyses?

Reply # 8: We appreciate the reviewer’s request for further clarification on this matter. For correlation analysis, Spearman’s rank correlation coefficient was used to assess the relationship between miRNA122 and traditional liver biomarkers (ALT, AST, and TBL). Given the small sample size, Spearman’s test was chosen due to being suitable for non-parametric data and less sensitive to outliers, making it appropriate for this type of longitudinal biomarker data. Regarding demographic and clinical factors, preliminary analyses did not reveal any significant differences in miRNA122 levels based on age, sex, or the severity/type of liver injury. However, due to the limited cohort size, these comparisons were exploratory, and larger studies will be needed to robustly assess these potential influences.

We acknowledge that confounding factors such as medication history and comorbidities could impact miRNA122 levels. However, given the study’s primary aim of evaluating the feasibility of the DCL PCR-Free method for miRNA122 detection, no adjustments for confounding factors were performed in the correlation analyses.

To further clarify these concerns, we added a new “statistical analysis section” the at the end of methods: “Statistical analyses were performed using GraphPad Prism (version 9.4.1, GraphPad Software, San Diego, CA, USA). To assess the relationship between miRNA122 levels and traditional liver biomarkers (ALT, AST, and TBL), Spearman’s rank correlation coefficient (r) was used. A p-value < 0.05 was considered statistically significant”.

**Question # 9:** How does the DCL PCR-Free method compare in sensitivity, specificity, and cost to other cutting-edge techniques such as droplet digital PCR (ddPCR) or next-generation sequencing (NGS) for miRNA detection? What are the barriers to translating this technology into routine clinical practice, such as cost, scalability, or regulatory approval? The figures are clear, but the calibration curve and technical details could be further elaborated.

Reply # 9: We are finalising the funding required to complete clinical validation, GMP manufacturing and enter the regulatory pathway to obtain CE-IVDR marking and FDA approval for our IVD. This project will start in summer 2025. We do not see any insurmountable barriers.

In our 2020 Analytical Chemistry paper, our DCL technology demonstrated superior performance compared to digital PCR in rat plasma samples. While the limit of detection (LOD) for our technology using calibrators in buffers may be inferior to PCR-based technologies, our real sample analysis has provided robust data. This is because PCR-based technologies require small RNA isolation, reverse transcription and PCR amplification of these sequences, which introduces potential problems with isomiRs. Our technology completely avoids these steps.

**Reviewer #2**

The manuscript entitled "miRNA122 interrogation via PCR-free method to track liver recovery" presents an innovative approach to detecting and monitoring microRNAs in patients with acute liver injury (ALI). The authors employ the Dynamic Chemical Labeling (DCL) PCR-Free method, demonstrating its sensitivity and precision in tracking miRNA122 levels during liver recovery. Their findings indicate a strong correlation between miRNA122 and traditional liver injury biomarkers, supporting the potential of this technique as a valuable diagnostic and tool.

While the study provides promising results and the method appears to be robust, several key issues need to be addressed before publication.

Major Concerns:

1. The figures in the manuscript are of extremely poor quality, making them illegible. Figure 1’s axis labels cannot be read, and Figure 2 appears as a black rectangle. Without properly formatted figures, it is impossible to validate the authors’ claims. The manuscript must be resubmitted with high-quality, legible figures to allow for proper assessment.

We sincerely apologize for the issues with Figure 1 and Figure 2 (Fig1 and Fig2) in the previous submission. We have now regenerated both figures with improved resolution and formatting to ensure they are fully legible and accurately represent our data.

The revised manuscript, including the updated figures, has been resubmitted for your consideration. We appreciate your time and the opportunity to address this issue.

Minor Concerns:

1. The results section should include an in silico characterization of miRNA122. Specifically, its genomic location, nearby genes, co-regulated elements, and predicted targets based on miRTarBase should be discussed. Additionally, the pathways regulated by miRNA122 should be incorporated into the discussion to provide further biological context.

Action # 1: We appreciate the reviewer’s suggestion to include an *in silico* characterization of miRNA122. However, the primary objective of this study was to evaluate the feasibility of the DCL PCR-Free method for miRNA122 quantification and its correlation with traditional liver biomarkers in ALI patients. Given this focus, an in-depth in silico analysis falls outside the intended scope of our study. That said, we recognize the importance of providing additional biological context for miRNA122 in the discussion. To address this, we have expanded the Discussion section to include the following information (between lines 235-245):

“*In particular, the miR122 plays a key role in the maintenance of liver homeostasis, participating in the regulation of differentiation processes, inflammation, lipid metabolism and apoptosis. However, the role of miR122 in liver diseases is not yet known in detail. Knock-down of miR122 in mice has been shown to favor the development of steatohepatitis, fibrosis and HCC [38, 39]. In the case of DILI by APAP, in vitro and in vivo models demonstrated that miR122 knock-down was protective against liver damage [40]. During hepatocyte damage or stress, miR122 is released into circulation, where it is relatively stable, which allows its detection as a non-invasive biomarker.*”

Three new references have been included respectively 38,39 and 40.

*38.	Xu H, He JH, Xiao ZD, Zhang QQ, Chen YQ, Zhou H, et al. Liver-enriched transcription factors regulate microRNA-122 that targets CUTL1 during liver development. Hepatology. 2010;52(4):1431-1442. doi:10.1002/hep.23818.*

*39.	Yang Z, Wu W, Ou P, Wu M, Zeng F, Zhou B, et al. miR-122-5p knockdown protects against APAP-mediated liver injury through upregulating NDRG3. Mol Cell Biochem. 2021;476(2):1257-1267. doi:10.1007/s11010-020-03988-0.*

*40.	Robles-Diaz M, Garcia-Cortes M, Medina-Caliz I, Gonzalez-Jimenez A, Gonzalez-Grande R, Navarro JM, et al. The value of serum aspartate aminotransferase and gamma-glutamyl transpeptidase as biomarkers in hepatotoxicity. Liver Int. 2015;35(11):2474-2482. doi:10.1111/liv.12834.*

The following references have been renumbered accordingly.

2. The manuscript contains excessive use of adjectives, particularly in the results and discussion sections. Precise numerical values should replace subjective descriptions. For example:

1. "Strong correlation" (Line 233) should include the specific correlation coefficient (R-value).
2. "These findings are interesting…" (Line 234) should be removed for conciseness.
3. "This milestone" (Line 257) should also be removed as it is overly subjective.
4. The discussion should be rewritten for clarity and conciseness, focusing on the key findings without unnecessary embellishment.

Given the significance of the study, I assume the figure issues are an honest mistake, but they must be corrected before the results can be properly reviewed. Addressing these concerns will significantly improve the clarity and impact of the manuscript.

Action # 2: We appreciate the reviewer’s feedback to help improve the manuscript. We agree that the Results and Discussion sections should be more concise and focused on presenting data objectively. To address this comment, we have:

5. Replaced "Strong correlation" (Lines 256-257) with "miRNA122 demonstrated a strong correlation with ALT (r = 0.8150) and ALT AST (r = 0.7895)."
6. Removed the phrase "These findings are interesting…"
7. Removed "This milestone" in the conclusion

We have also revised the Discussion to focus on key findings, ensuring that conclusions are clearly supported by data. Redundant or overly embellished phrases have been removed.
